# Association between PD-L1 expression combined with tumor-infiltrating lymphocytes and the prognosis of patients with advanced hypopharyngeal squamous cell carcinoma

**DOI:** 10.18632/oncotarget.21564

**Published:** 2017-10-06

**Authors:** Takeharu Ono, Koichi Azuma, Akihiko Kawahara, Tetsuro Sasada, Satoshi Hattori, Fumihiko Sato, Buichiro Shin, Shun-Ich Chitose, Jun Akiba, Umeno Hirohito

**Affiliations:** ^1^ Department of Otolaryngology-Head and Neck Surgery, Kurume University School of Medicine, Kurume, Japan; ^2^ Division of Respirology, Neurology, and Rheumatology, Department of Internal Medicine, Kurume University School of Medicine, Kurume, Japan; ^3^ Department of Diagnostic Pathology, Kurume University Hospital, Kurume, Japan; ^4^ Cancer Vaccine Center, Kanagawa Cancer Center Research Institute, Yokohama, Japan; ^5^ Biostatistics Center, Kurume University School of Medicine, Kurume, Japan

**Keywords:** PD-L1, immunohistochemistry, hypopharyngeal squamous cell carcinoma, neoadjuvant chemotherapy, CD8+ TILs

## Abstract

Limited information is available regarding the immune-related prognostic factors of patients with advanced hypopharyngeal squamous cell carcinoma (HPSCC). The expression of programmed cell death-ligand 1 (PD-L1) in tumor cells contributes to a mechanism that allows cancer cells to escape immune surveillance. We investigated whether PD-L1 or human leukocyte antigen (HLA) class I expression in tumor cells and the tumor-infiltrating lymphocyte (TIL) density were associated with the tumor response to neoadjuvant chemotherapy (NAC) and survival in patients with advanced HPSCC. We retrospectively reviewed 83 consecutive patients with stage III or IV HPSCC who received NAC. We evaluated PD-L1 and HLA class I expression and TIL density using immunohistochemistry. Univariate and multivariate analyses demonstrated that CD8^+^ TIL density was an independent and significant predictive factor for the response to NAC, progression-free survival (PFS) and overall survival (OS), whereas PD-L1 or HLA class I expression did not significantly correlate. The subgroup analysis revealed that a higher CD8^+^ TIL density without detectable PD-L1 expression tended to be associated with longer patient survival. These results suggest that PD-L1 expression levels combined with CD8^+^ TIL density may serve as a predictive biomarker for patients with stage III or IV HPSCC receiving NAC.

## INTRODUCTION

Hypopharyngeal squamous cell carcinoma (HPSCC) accounts for 3%–5% of all head and neck squamous cell carcinoma (HNSCC) [1]. Most patients with HPSCC present with advanced disease and have the worst prognosis among those with HNSCCs arising from different anatomical sites, despite the development of treatments comprised of surgery and chemotherapy with or without radiotherapy [2].

Although neoadjuvant chemotherapy (NAC) has no clear evidence of survival benefits [3], it has been used as a treatment option for larynx-preservation in locally advanced laryngeal and hypopharyngeal cancer [4, 5]. Despite the correlation between tumor responses to NAC and patient prognosis [6, 7], few reports describing the predictive biomarkers for tumor responses to NAC are available in the context of HNSCC [8, 9].

The CD28 protein family member, programmed cell death 1 (PD-1), is a receptor expressed on the surface of T cells, which suppresses their activation and proliferation. The binding of PD-1 to its ligand, programmed cell death-ligand 1, induces apoptosis or exhaustion in activated T cells, and inhibition of this interaction enhances the antitumor activity of T cells [10]. Therefore, PD1 and PD-L1 are considered immune checkpoint molecules. PD-L1 is frequently overexpressed in many types of human cancers [10], and therefore, the PD-1/PD-L1 axis is considered to contribute to a mechanism that allows cancers to escape immune surveillance. Specific antibodies, which act as immune checkpoint blockers to inhibit the interactions between PD-1 and its ligands, PD-L1 and PD-L2, exhibit promising clinical efficacy against certain malignancies [11–13].

Currently, there is limited information available regarding the prognostic values of PD-L1 or HLA class I expression and TILs in patients with HPSCC receiving NAC. Therefore, this study aimed to evaluate the predictive relevance of these factors in patients with advanced HPSCC who received NAC.

## RESULTS

### Patient characteristics

The clinical characteristics of the 83 patients included in this study are presented in Table 1. The median age of the patients at diagnosis was 65 years (range: 40–80 years). A total of 79 patients (95%) were men, and 75 (90%), 6 (7%), and 2 (2%) patients had PS (0), PS (1), and PS (2), respectively. Mild (< 40 packs of cigarettes per year) and heavy (≥ 40 packs of cigarettes per year) smokers consisted of 33 (40%) and 45 (54%) patients, respectively. Moderate (10-30 g/day) and heavy (≥ 30 g/day) alcohol consumers consisted of 38 (46%) and 40 (48%) patients, respectively. Tumor histology was classified as well differentiated (30 patients), moderately differentiated (42 patients), or poorly differentiated (11 patients). The anatomical subsites were classified as pyriform sinus (70 patients), post-cricoid (6 patients), and posterior wall (7 patients). In accordance with the system adopted by the Union for International Cancer Control (UICC) TNM Classification of Malignant Tumors 7^th^ edition [14], 16 (19%), 55 (66%), and 12 (15%) patients, respectively exhibited stage III, IVA, or IVB disease at the time of diagnosis.

**Table 1:** Patients’ characteristics

Characteristics		N (%)
Age (years) (*n* = 83)		
	Median	65
	Range	40–80
Performance status		
	0	75 (90)
	1	6 (7)
	2	2 (2)
Sex		
	Male	79 (95)
	Female	4 (5)
Follow up (month)		
	Median	32
	Range	2–127
Progression-free survival(month)		
	Median	24
	Range	1–127
Overall survival (month)		
	Median	32
	Range	2–127
Current status		
	Dead	48 (58)
	Alive	35 (42)
Smoking status		
	Never	5 (6)
	Mild	33 (40)
	Heavy	45 (54)
Alcohol status		
	Never	5 (6)
	Moderate	38 (46)
	Heavy	40 (48)
Differentiation		
	Well differentiated	30 (36)
	Moderately differentiated	42 (51)
	Poorly differentiated	11 (13)
Subsite		
	Pyriform sinus	70 (84)
	Post cricoid	6 (7)
	Posterior wall	7 (8)
T stage		
	T1	0
	T2	15 (18)
	T3	38 (46)
	T4	30 (36)
N Stage		
	N0	10 (12)
	N1	13 (16)
	N2	49 (59)
	N3	11 (13)
Stage		
	III	16 (19)
	IVA	55 (66)
	IVB	12 (15)
NAC categories		
	PF	45 (54)
	TPF	38 (46)
Response to NAC		
	Complete response	0
	Partial response	39 (47)
	Stable disease	34 (41)
	Progressive disease	10 (12)
Definitive treatment		
	Surgery	48 (58)
	CCRT	29 (35)
	Palliation	6 (7)
Postoperative treatment		
	None	20 (24)
	RT	20 (24)
	CCRT	8 (10)
HLA class I expression		
	< 25%	35 (42)
	25–74	34 (41)
	≥ 75%	14 (17)
PD-L1 TPS		
	< 1%	57 (68)
	1%–49%	13 (16)
	≥ 50%	13 (16)

NAC, neoadjuvant chemotherapy; PF, cisplatin and 5-fluorouracil; TPF, docetaxel, cisplatin, and 5-fluorouracil; TPS, tumor proportion score; CCRT, concurrent chemoradiotherapy.

### Treatment protocol

The various treatments and associated tumor responses are shown in Table 1. All patients received platinum and fluorouracil-based agents as NAC as for the initial treatment. A total of 45 patients received intravenous PF (20 mg/m^2^ cisplatin on days 1-5 and 1000 mg/m^2^ 5-fluorouracil on days 1-5 in one cycle) and 38 patients received TPF (60 mg/m^2^ docetaxel on day1, 60 mg/m^2^ cisplatin on day 1, and 700 mg/m^2^ 5-fluorouracil on days 1-4 in one cycle). An evaluation of the tumor response to NAC based on the response evaluation criteria in solid tumors, was performed using computed tomography and magnetic resonance imaging. Partial responses (PR), stable disease (SD), and progressive disease (PD) were observed in 39 (47%), 34 (41%), and 10 (12%) patients, respectively. Six patients (7%) received palliative treatment because of rapid locoregional progression or the appearance of distant metastasis, 29 patients (35%) received concurrent chemoradiotherapy (CCRT) containing platinum and 5-fluorouracil-based agents, and 48 patients (58%) received surgical treatment (e.g., transoral resection or total pharyngolaryngectomy) combined with a neck dissection. Twenty (24%) and eight (10%) patients received RT and CCRT, respectively, as a post-operative treatment. Post-operative RT for primary and regional sites consisting of a single daily irradiation administered at 1.8 Gy per fraction (total: 60-61Gy), was initiated 3–4 weeks after surgery. Definitive or post-operative CCRT containing cisplatin (5 mg/m^2^ on days 1-15) and 5-fluorouracil (250 mg/m^2^ on days 115) was performed with a daily irradiation administered at 1.8 Gy per fraction (total: 61-71Gy).

### PD-L1 or HLA class I expression and CD3^+^, CD4^+^, or CD8^+^ TIL density

**Figure 1:**
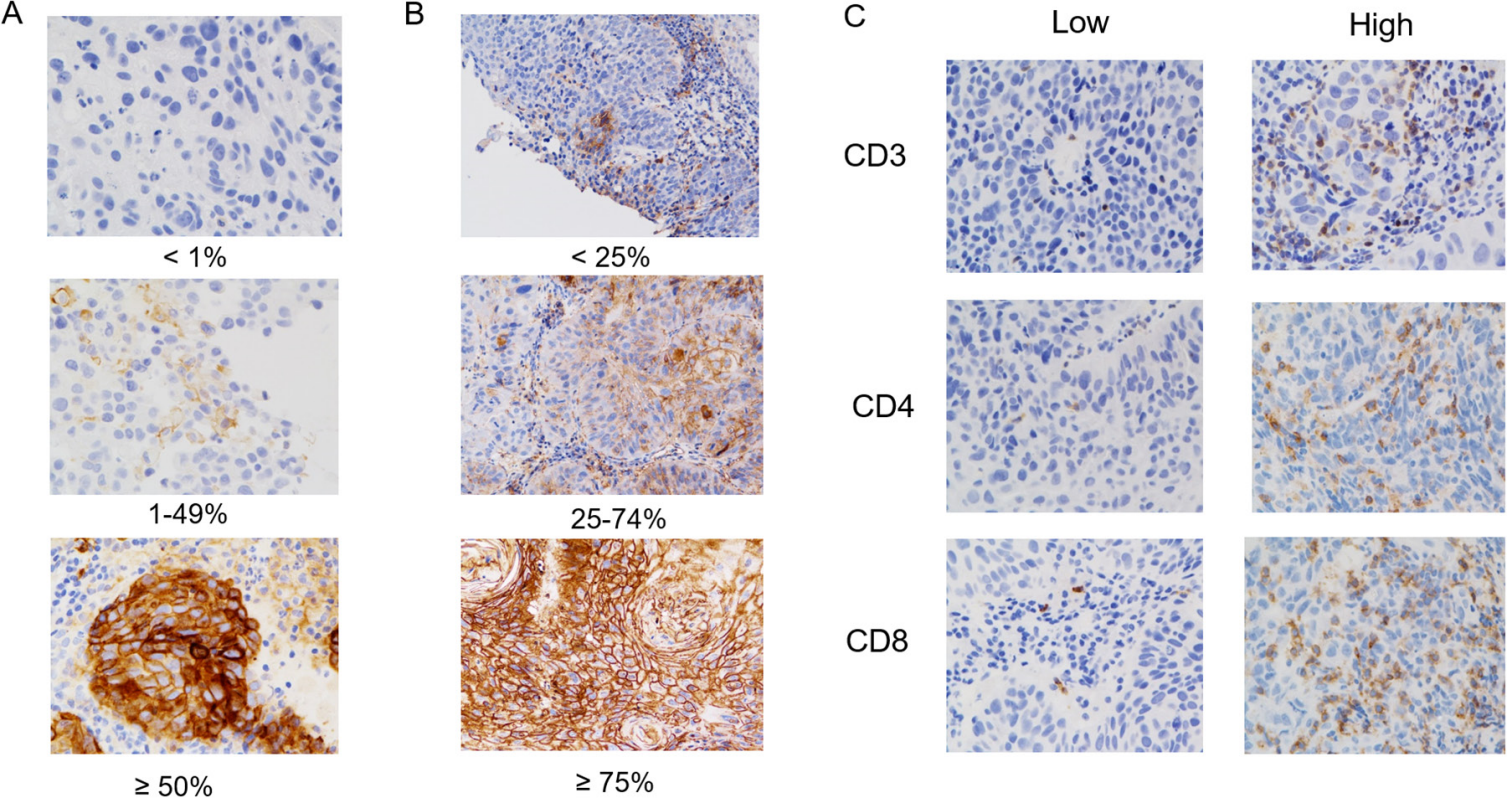
Immunohistochemical (IHC) staining patterns of PD-L1, HLA class I, CD3, CD4, and CD8 of patients with advanced hypopharyngeal squamous cell carcinoma Representative examples of patients whose tumor proportion scores (TPS) were classified as < 1%, 1%–49%, and ≥ 50% for the membrane expression of PD-L1 are shown **(A)**. The level of HLA class I expression was classified as < 25%, 25%–74%, and ≥ 75% **(B)**. High or low IHC staining patterns for CD3^+^, CD4^+^, and CD8^+^ TILs are shown **(C)**.

We restricted our immunohistochemical analysis to PD-L1 or HLA class I expression and CD3^+^, CD4^+^, or CD8^+^ TIL density due to limited tissue availability. Figure 1 shows the representative staining patterns of PD-L1, HLA class I, CD3, CD4, and CD8 in the tumor specimens. PD-L1 expression was observed in the membrane, cytoplasm, or both in tumor cells and/or stromal lymphocytes. PD-L1 expression in the tumors [tumor proportion score (TPS) ≥1%] was detected in 26 (31%) patients. Regarding the expression of HLA class I in tumor cells, positive (expression level ≥ 75%), heterogeneous (expression level: 25%-74 %), and negative (expression level < 25%) staining was detected in 14 (17%), 34 (41%), and 35 (42%) of patients, respectively (Table 1). The median number of CD3^+^, CD4^+^, and CD8^+^ TILs were 50 (range: 0–383), 21 (range: 0–186), and 36 (range: 0–347), respectively; these values were used to discriminate patients with high or low TIL density. Although we examined the expression of P16, which was associated with HPV infection, using immunohistochemistry, the CD8^+^ TIL density was not correlated with the level of P16 expression (data not shown).

### Correlation between PD-L1 or HLA class I expression and patient characteristics

The relationships between PD-L1 or HLA class I expression and patient demographics are presented in Table 2. No significant correlations were not detected between PD-L1 expression and age (p = 0.479), smoking status (p = 1.000), alcohol consumption (p = 0.488), performance status (p = 0.710), tumor differentiation (p = 0.139), T classification (p = 1.000), or N classification (p = 0.605). The correlation between PD-L1 and HLA class I expression was significant (p = 0.030); however, PD-L1 expression was not significantly associated with the CD3^+^ TIL density (p = 1.000). In addition, PD-L1 expression tended to be positively correlated with CD4^+^ (p = 0.061) and CD8^+^ (p = 0.061) TIL density, albeit this was not statistically significant. Of note, significant correlations were found between PD-L1 expression and CD4^+^ (p = 0.010) or CD8^+^ (p = 0.022) TIL density when these TIL densities were analyzed as continuous variables (Supplementary Figure 1).

**Table 2:** Relationship between PD-L1 or HLA class I expression and patients’ characteristics

Characteristic	N	PD-L1			HLA class I		
		Negative	Positive	*P* value^a^	Low	High	*P* value^a^
	83	57	26		69	14	
Age (years)							
≤ 65	42	27	15	0.479	36	6	0.570
> 65	41	30	11		33	8	
Smoking status							
Never or Mild	38	26	12	1.000	29	9	0.151
Heavy	45	31	14		40	5	
Alcohol status							
Never or Moderate	43	28	15	0.488	35	8	0.773
Heavy	40	29	11		34	6	
Performance status							
0	75	52	23	0.710	63	12	0.617
1 or 2	8	5	3		6	2	
Differentiation							
Well	30	24	6	0.139	25	5	1.000
Moderately or Poorly	53	33	20		44	9	
T classification							
T2	15	10	5	1.000	12	3	0.711
T3–4	68	47	21		57	11	
N classification							
N0–1	23	17	6	0.605	18	5	0.518
N2–3	60	40	20		51	9	
HLA class I							
High	14	6	8	0.030	-	-	-
Low	69	51	18		-	-	
CD3							
High	41	28	13	1.000	30	11	0.020
Low	42	29	13		39	3	
CD4							
High	41	24	17	0.061	32	9	0.245
Low	42	33	9		37	5	
CD8							
High	41	24	17	0.061	30	11	0.020
Low	42	33	9		39	3	

^a^Fisher's exact test.

Regarding the correlation with HLA class I expression, significant differences were not detected for age (p = 0.570), smoking status (p = 0.151), alcohol consumption (p = 0.773), performance status (p = 0.617), tumor differentiation (p = 1.000), T classification (p = 0.711), N classification (p = 0.518), or CD4^+^ TIL density (p = 0.245). In contrast, CD3^+^ or CD8^+^ TIL density was significantly correlated with HLA class I expression (p = 0.020 and p = 0.020, respectively).

### Correlation between the tumor response to NAC and patient characteristics

Patients who exhibited a partial response (PR) to NAC were defined as responders, whereas those with stable disease (SD) or progressive disease (PD) were defined as non-responders. As shown in Supplementary Figure 2, the responders to NAC were associated with a significantly better prognosis than the non-responders. The relationships between the response rate (RR) to NAC and patient demographics are presented in Table 3. In the univariate analysis, N classification (RR: N0-N1vs. N2-3, 65.2% vs. 40%; p = 0.043), CD3^+^ TIL density (RR: high vs. low, 58.5% vs. 35.7%; p = 0.039), and CD8^+^ TIL density (RR: high vs. low, 63.4% vs. 31.0%; p = 0.004) were significantly correlated with the tumor response to NAC. HLA class I expression was not included in the multivariate analysis because it was significantly correlated with PD-L1 expression, CD3^+^ TIL density, or CD8^+^ TIL density (p = 0.030, 0.020, and 0.020, respectively). The multivariate analysis demonstrated that N classification (odds ratio [OR]: 0.29; 95% confidence interval [CI]: 0.09–0.89; p = 0.031) and CD8^+^ TILs density (OR: 0.32; 95%CI: 0.11–0.95; p = 0.039) were significant factors for the tumor response to NAC.

**Table 3:** Univariate and multivariate analyses of clinicopathologic factors associated with response to NAC

Factor	N	Response rate (%)	Univariate	Multivariate
			*P* value^a^OR (95% CI)	*P* value^a^OR (95% CI)
Age (years)				
≤ 65	42	56.1	0.102	0.154
> 65	41	38.1	2.07 (0.86–4.99)	2.02 (0.77–5.33)
Smoking status				
Never or Mild	38	39.5	0.209	
Heavy	45	53.3	1.75 (0.73–4.20)	
Alcohol status				
Never or Moderate	43	48.8	0.726	
Heavy	40	45.0	0.86 (0.36–2.03)	
Performance status				
0	75	48.0	0.574	
1 or 2	8	37.5	0.65 (0.15–2.92)	
Differentiation				
Well	30	50.0	0.679	
Moderately or poorly	53	45.3	1.21 (0.49–2.96)	
T classification				
T2	15	46.7	0.978	0.628
T3-4	68	47.1	1.02 (0.33–3.11)	0.73 (0.20–2.60)
N classification				
N0-1	23	65.2	0.043	0.031
N2-3	60	40.0	0.36 (0.13–0.98)	0.29 (0.09–0.89)
NAC categories				
PF	45	48.9	0.706	
TPF	38	44.7	0.84 (0.36–2.01)	
HLA class I				
High	14	57.1	0.407	
Low	69	44.9	0.61 (0.19–1.95)	
PD-L1				
Positive	26	53.8	0.399	0.591
Negative	57	43.9	0.67 (0.26–1.70)	0.73 (0.24–2.26)
CD3				
High	41	58.5	0.039	0.378
Low	42	35.7	0.39 (0.16–0.95)	0.61 (0.20–1.82)
CD4				
High	41	51.2	0.446	0.711
Low	42	42.8	0.71 (0.30–1.70)	0.81 (0.28–2.39)
CD8				
High	41	63.4	0.004	0.039
Low	42	31.0	0.26 (0.10–0.64)	0.32 (0.11–0.95)

^a^Logistic regression analysis; OR, odds ratio; CI, confidence interval; NAC, neoadjuvant chemotherapy; PF, cisplatin and 5-fluorouracil; TPF, docetaxel, cisplatin, and 5-fluorouracil.

### Survival analysis

**Figure 2:**
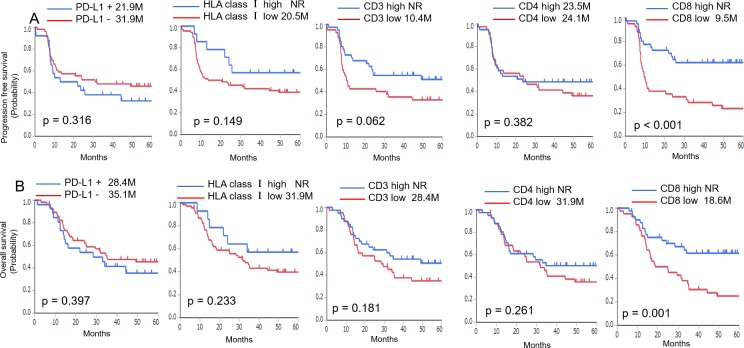
Kaplan-Meier analysis of the progression-free survival (PFS) **(A)** and overall survival (OS) **(B)** of patients with advanced hypopharyngeal squamous cell carcinoma exhibiting positive or negative expression of PD-L1, HLA class I, and high or low CD3^+^, CD4^+^, and CD8^+^ TIL density. Significant differences were evaluated using a log-rank test.

At the time of analysis, the median follow-up time for all patients was 32 months, and 35 patients (42%) were alive at the time of the last follow-up (Table 1). The median progression-free survival (PFS) and overall survival (OS) were 24 and 32 months, respectively, and PFS and OS rates at 60 months were 41.9% and 42.5%, respectively. A Kaplan-Meier analysis was performed to evaluate whether PD-L1 or HLA class I expression and CD3^+^, CD4^+^, or CD8^+^ TIL density were associated with PFS or OS (Figure 2). The expression of PD-L1 or HLA class I was not significantly correlated with PFS (positive vs negative: median 21.9 vs. 31.9 months, p = 0.316; high vs. low: median not reached [NR] vs. 20.5 months; p = 0.149; respectively] or OS (positive vs. negative: median 28.4 vs. 35.1 months, p = 0.397; high vs. low: median NR vs. 31.9 months, p = 0.233; respectively). Although the differences were not significant, patients with higher numbers of CD3^+^ TILs tended to have a longer PFS (high vs. low: median NR vs. 10.4 months; p = 0.062), but not OS (high vs. low: median NR vs. 28.4 months; p = 0.181). CD4^+^ TIL density did not exhibit a significant correlation with PFS (high vs. low: median 23.5 vs. 24.1 months, p = 0.382) or OS (high vs. low: median NR vs. 31.9 months, p = 0.261). In contrast, patients with higher numbers of CD8^+^ TILs experienced significantly longer PFS (high vs. low: median NR vs. 9.5 months; p < 0.001) and OS (high vs. low: median NR vs. 18.6 months; p = 0.001). Furthermore, the univariate analysis indicated that N classification was a significant predictive factor of OS (hazard ratio [HR]: 0.47; 95%CI: 0.21–0.93; p = 0.029) (Table 4).

**Table 4:** Univariate and multivariate analyses of clinicopathologic factors associated with PFS and OS

Factor	N	PFS		OS	
		Univariate	Multivariate	Univariate	Multivariate
		*P* value^a^HR (95% CI)	*P* value^a^HR (95% CI)	*P* value^a^HR (95% CI)	*P* value^a^HR (95% CI)
Age (years)					
≤ 65	42	0.521		0.743	
> 65	41	1.20 (0.68–2.12)		1.10 (0.62–1.95)	
Smoking status					
Never or Mild	38	0.666		0.345	
Heavy	45	1.13 (0.64–1.99)		1.31 (0.74–2.32)	
Alcohol status					
Never or Moderate	43	0.461		0.679	
Heavy	40	0.81 (0.46–1.42)		0.89 (0.50–1.56)	
Performance status					
0	75	0.276		0.188	0.298
1 or 2	8	0.60 (0.28–1.58)		0.53 (0.25–1.41)	0.60 (0.26–1.65)
Differentiation					
Well	30	0.672		0.943	
Moderately or poorly	53	1.13 (0.62–2.01)		1.02 (0.56–1.82)	
T classification					
T2	15	0.464	0.581	0.437	0.602
T3–4	68	1.29 (0.63–2.45)	1.21 (0.58–2.34)	1.31 (0.64–2.50)	1.21 (0.57–2.40)
N classification					
N0–1	23	0.060	0.102	0.029	0.048
N2–3	60	0.53 (0.25–1.02)	0.56 (0.26–1.11)	0.47 (0.21–0.93)	0.49 (0.22–0.99)
NAC categories					
PF	45	0.263		0.417	
TPF	38	0.72 (0.41–1.28)		0.78 (0.44–1.41)	
HLA class I					
High	14	0.127		0.209	
Low	69	0.54 (0.20–1.17)		0.60 (0.22–1.30)	
PD-L1					
Positive	26	0.326	0.070	0.405	0.073
Negative	57	1.35 (0.73–2.43)	1.85 (0.95–3.48)	1.30 (0.42–1.45)	1.86 (0.94–3.57)
CD3					
High	41	0.063	0.437	0.181	0.596
Low	42	0.59 (0.33–1.03)	0.78 (0.41–1.46)	0.67 (0.38–1.20)	0.84 (0.44–1.58)
CD4					
High	41	0.385	0.517	0.260	0.301
Low	42	0.78 (0.44–1.37)	0.78 (0.42–1.44)	0.72 (0.40–1.27)	0.72 (0.38–1.34)
CD8					
High	41	< 0.001	0.001	0.001	0.005
Low	42	0.34 (0.18–0.61)	0.33 (0.16–0.62)	0.38 (0.20–0.70)	0.38 (0.19–0.75)

^a^Cox proportional hazards model. PFS, progression-free survival; OS, overall survival; CI, confidence interval; NAC, neoadjuvant chemotherapy; PF, cisplatin and 5-fluorouracil; TPF; docetaxel, cisplatin, and 5-fluorouracil.

The multivariate analyses revealed that the density of CD8^+^ TILs was an independent and significant predictive factor of PFS (HR: 0.33; 95%CI: 0.16–0.62; p = 0.001) and OS (HR: 0.38; 95%CI: 0.19–0.75; p = 0.005) (Table 4). In addition, the multivariate analysis revealed that N classification was a significant factor associated with OS (HR: 0.49; 95%CI: 0.22–0.99; p = 0.048 (Table 4).

### Correlation between survival and PD-L1 expression combined with CD8^+^ TIL density

**Figure 3:**
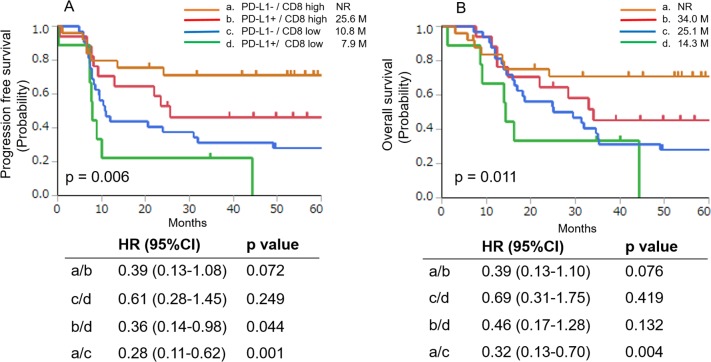
Kaplan-Meier analysis of PFS **(A)** and OS **(B)** of patients with positive or negative PD-L1 expression combined with high or low CD8^+^ TIL density. Significant differences were evaluated using a log-rank test.

We performed subanalyses to determine whether the combination of PD-L1 expression and CD8^+^ TIL density could predict patient prognosis. For this purpose, patients were divided into four subgroups, which were defined by PD-L1 expression and CD8^+^ TIL density [15, 16]. A Kaplan-Meier analyses of PFS and OS in the four subgroups (PD-L1^−^/CD8^high^, PD-L1^+^/CD8^high^, PD-L1^−^/CD8^low^, and PDL1^+^/CD8^low^) are presented in Figure 3. The median PFS values were NR in the PD-L1^−^/CD8^high^ group, 25.6 months in the PD-L1^+^/CD8^high^ group, 10.8 months in the PD-L1^−^/CD8^low^ group, and 7.9 months in the PD-L1^+^/CD8^low^ group (p = 0.006) (Figure 3A). The univariate analysis revealed that the PFS of the CD8^high^ group was longer than that of the CD8^low^ group in patients negative or positive for PD-L1 expression (PD-L1^+^/CD8^high^ vs PD-L1^+^/CD8^low^, p = 0.044; PD-L1^−^/CD8^high^ vs PD-L1^−^/CD8^low^ groups, p = 0.001, respectively). In addition, although not statistically significant, the PFS of the PD-L1^−^/CD8^high^ group also tended to be longer than that of the PD-L1^+^/CD8^high^ group (p = 0.072).

The median OS was NR in the PD-L1^−^/CD8^high^ group, 34.0 months in the PD-L1^+^/CD8^high^ group, 25.1 months in the PD-L1^−^/CD8^low^ group, and 14.3 months in the PD-L1^+^/CD8^low^ group (p = 0.011) (Figure 3B). The univariate analysis revealed that the OS of the CD8^high^ group was longer than that of the CD8^low^ group in patients with negative or positive PD-L1 expression (PD-L1^+^/CD8^high^ vs. PD-L1^+^/CD8^low^, p = 0.132 and PD-L1^−^/CD8^high^ vs. PD-L1^−^/CD8^low^ groups, p = 0.004, respectively). In addition, although not statistically significant, the OS of the PD-L1^−^/CD8^high^ group also tended to be longer than that of the PD-L1^+^/CD8^high^ group (p = 0.076).

## DISCUSSION

CD8+ cytotoxic T cells are a crucial component of the cellular immune system and play a pivotal role in cell-mediated antitumor immune responses [17, 18]. For example, patients with HNSCC who exhibit a greater number of tumor-infiltrating CD8+ TILs are reported to survive longer [9, 19–21]. Consistent with these studies, we this study demonstrated that a higher CD8^+^ TIL density was significantly associated with long-term survival, indicating that CD8^+^ T cells may play an important role in antitumor immunity in HPSCC patients who received NAC [22]. In addition, the subgroup analysis suggested that further stratification based on PD-L1 expression could be useful for predicting the prognosis of patients with a higher CD8^+^ TIL density. This suggests that a co-assessment of CD8^+^ TIL density and PD-L1 expression may be a predictive biomarker for patients with HPSCC receiving NAC.

Several immune-related biomarkers used to predict chemotherapeutic responses have been identified for various types of cancers [23]. For example, a higher CD3^+^ TIL density and fewer intraepithelial macrophages in tumors have been found to exhibit a better response to NAC in patients with head and neck cancer [9]. In the present study, the multivariate analysis revealed that CD8^+^ TIL density was significantly associated with tumor response to NAC. To our knowledge, there have been no published reports demonstrating that CD8^+^ TIL density may be a useful biomarker for predicting tumor responses to NAC in patients with advanced HPSCC. Because chemotherapy has been reported to change the tumor microenvironment via high antigen exposure and the accumulation of dendritic cells, which stimulate CD8^+^ T cells and the type I IFN pathway [24], pre-existing CD8^+^ TILs may be one of the key components for efficient responses to NAC.

Negative regulatory mechanisms (e.g., increased PD-1 expression) may impact the prognostic importance of CD8^+^ TILs. For example, previous reports have demonstrated that patients with high PD-1^+^/CD8^+^ TILs were associated with a significantly worse prognosis compared to those with low PD-1^+^/CD8^+^ TILs [25, 26]. These findings suggest that increased PD-1 expression on CD8^+^ TILs may represent an exhausted state of effector cells, thus resulting in a reduced anti-tumor effect. Therefore, it may be important to investigate the level of PD-1 expression on CD8^+^ TILs to distinguish between functional and exhausted CD8^+^ TILs. However, unfortunately, we were unable to examine the PD-1 expression in TILs using immunohistochemistry in this study due to the limited number of available tumor tissues for immunohistochemistry. We would like to investigate the significance of the PD-1 expression on CD8^+^ TILs in patients with advanced HPSCC in future studies and to report the results in detail in future publications.

Although blockade of the PD-1/PD-L1 pathway using monoclonal antibodies is a novel therapeutic strategy for treating patients with recurrent HNSCC [27], the association between PD-L1 expression and the prognosis of patients with HNSCC has not been well described. Recent studies have demonstrated that higher levels of PD-L1 expression are associated with a poor prognosis in various types of cancers [28–32], whereas other studies have presented controversial results [33–35]. For example, PD-L1 expression was reportedly not associated with patient prognosis in laryngeal and pharyngeal squamous cell carcinoma [9, 36]. Consistent with these results, the present study did not find a significant correlation between PD-L1 expression and the prognosis of patients with stage III or IV HPSCC who received NAC.

It has been reported that IFN-γ produced by tumor-specific TILs could induce PD-L1 expression in tumor cells and/or immune cells as an adaptive immune response. Nevertheless, because CD8^+^ TIL density does not strictly correlate positively with PD-L1 expression, the PD-1/PD-L1 axis may function as an immune escape mechanism only in some (but not all) patients; albeit the precise mechanism remains unclear. Therefore, we considered that an evaluation of CD8^+^ TIL density could become more meaningful when interpreted in conjunction with PD-L1 expression on tumor cells. The Kaplan-Meier analysis demonstrated that the four subgroups defined by PD-L1 expression and CD8^+^ TIL density demonstrated prognostic relevance. Of note, the PD-L1^+^/CD8^high^ group tended to exhibit a worse prognosis than the PD-L1^−^/CD8^high^ group. In the PD-L1^+^/CD8^high^ group, PD-L1 expression may be intrinsically expressed or efficiently induced as an adaptive immune escape response, possibly resulting in a worse prognosis. In contrast, in the PD-L1^−^/CD8^high^ group, immune suppressive mechanisms other than the PD-1/PD-L1 axis [e.g., regulatory T cells, tumor-associated macrophages, or myeloid-derived suppressor cells (MDSCs)], may be activated for the purpose of immune escape [37, 38]; however, the specific details remain unclear. Based on our finding that the PD-L1^−^/CD8^high^ group tended to have a better prognosis than the PD-L1^+^/CD8^high^ group, it may be possible that PD-L1 expression could induce more efficient immune suppression than other immune escape mechanisms. Consistent with the current study, previous studies have demonstrated similar results in other tumor types (e.g., lung and gastric cancer) [16, 37].

The anti-PD-L1 antibody MPDL3280A has been reported to elicit improved clinical responses in patients with tumors that express high levels of PD-L1 and contain higher numbers of tumor-infiltrating immune cells [39]. Similarly, it has been suggested that patients with tumor cells expressing PD-L1, as well as a high density of CD8^+^ TILs may achieve better outcomes via an anti-PD-1 blocking antibody [38]. Further studies are required to determine the predictive relevance of a co-assessment of PD-L1 expression and CD8^+^ TIL density for selecting patients with HPSCC who are most likely to respond to the PD-1/PD-L1 blocking treatments.

The current retrospective study has several limitations. First, the definitive treatments of the patients following NAC were heterogeneous. Second, the tissue samples obtained using nasal endoscopy were small, and may not reflect the entire tumor. Because the expression of PD-L1 on tumor cells, as well as the TIL density are often heterogeneous even within the same tumors, an accurate evaluation of PD-L1 expression and TIL density in small biopsy samples may occasionally be difficult in this study. Third, the threshold for positive immunohistochemistry results was not formally defined, and reproducibility was not formally assessed. Therefore, future investigations and validations are warranted to harmonize and standardize testing for PD-L1 expression using an immunohistochemical analysis of larger sample sizes.

In conclusion, the current study demonstrated that a higher CD8^+^ TIL density was significantly associated with the tumor responses to NAC and long-term survival in patients with advanced HPSCC who received NAC. In addition, our subanalysis suggested that patients who exhibited a higher CD8^+^ TIL density without detectable levels of PD-L1 in tumor cells survived longer. We propose that a co-assessment of CD8^+^ TIL density and PD-L1 expression would have greater predictive potential compared with an evaluation of each factor alone in patients with advanced HPSCC.

## MATERIALS AND METHODS

### Patients

We retrospectively screened consecutive patients diagnosed with stage III or IV HPSCC at Kurume University Hospital between 2000 and 2013. The inclusion criteria were as follows: pathological diagnosis of HPSCC; NAC treatment followed by surgery, definitive concurrent chemoradiotherapy (CCRT) with combined platinum-containing chemotherapy, or palliation; and the availability of adequate histological specimens containing tumor cells. The present study was conducted in accordance with the provisions of the Declaration of Helsinki and was approved by the Institutional Review Boards of Kurume University Hospital.

### Immunohistochemical analysis

We used 4-μm-thick sections of formalin-fixed, paraffin-embedded tissues. The sections were mounted on glass slides and then incubated with an anti-rabbit monoclonal antibody against PD-L1 (clone D3; Cell Signaling Technology, Danvers, MA) using a BenchMark ULTRA (Ventana Automated Systems, Inc., Tucson, AZ). Briefly, each slide was heated using the Ventana CC1 retrieval solution for 30 min and incubated with the PD-L1 antibody for 30 min. This automated system used the ultraVIEW DAB detection kit with 3, 3′ diaminobenzidine (DAB) as the chromogen (Ventana Automated Systems).

Immunohistochemical analysis of HLA class I (EMR8-5; ab70328; Abcam, Cambridge, England), CD3, CD4, and CD8 expression (Leica Microsystems, Newcastle-upon-Tyne, UK) was performed using a fully automated Bond-III system (Leica Microsystems). Briefly, each slide was treated using onboard heat-induced antigen retrieval with an epitope retrieval solution 2 for 10 min at 99°C, incubated with the antibody for 30 min at room temperature, and then incubated with a secondary antibody for 30 min at room temperature. This automated system used a Refine polymer detection kit with HRP (horseradish peroxidase)-polymer as the secondary antibody in conjunction with DAB.

All immunohistochemical analyses were evaluated by two observers (A.K. and J.A.) who were unaware of the patients’ condition. Disagreements in data interpretation were reviewed jointly, and a single consensus category was established. All patient samples for PD-L1 expression were divided into groups based on the TPS of < 1%, 1%–49%, and ≥ 50%, as detailed previously [40, 41]. Patients with PD-L1 TPS of ≥ 1% were considered positive, as described previously [42]. Regarding the expression of HLA class I, we divided the expression levels into three criteria as previously described: 1) positive, expressed on more than 75% of tumor cells; 2) heterogeneous, expressed between 25% and 74%; and 3) negative, expressed on less than 25% of tumor cells [43, 44]. Patients with positive HLA class I expression (≥75%) were defined as the high group. Furthermore, the TIL numbers were counted, and scoring was performed using a four-tier scale. We determined the median values of CD3^+^, CD4^+^, and CD8^+^ TILs to define the cut-off value for TIL density.

### Statistical analyses

Correlations between PD-L1 or HLA class I expression and the patient characteristics were analyzed using Fisher's exact test for categorical variables. We evaluated whether variables, including PD-L1 expression, were associated with the survival of patients with stage III or IV HPSCC who received NAC, followed by definitive treatment or best supportive care. To examine the predictive factors for the NAC response, univariate and multivariate logistic regression analyses were performed. The progression-free survival (PFS) and overall survival (OS) were calculated from the date of initiating NAC to tumor relapse (locoregional recurrence, distant metastasis, or both) or death, respectively. Kaplan–Meier analysis was used to assess patient's survival, and a log-rank test was used to evaluate the significant differences between and among two or four groups, respectively. Multivariate regression analysis was performed using a Cox proportional hazards model. All tests were two-sided, and *p* < 0.05 indicates a statistically significant difference. The statistical analyses were conducted using JMP version 11 (SAS Institute Inc., Cary, NC).

## SUPPLEMENTARY MATERIALS FIGURES



## Abbreviations

HPSCC: hypopharyngeal squamous cell carcinoma
PD-L1: programmed cell death-ligand 1
HLA: human leukocyte antigen
TIL: tumor-infiltrating lymphocyte
NAC: neoadjuvant chemotherapy
PD-1: programmed cell death 1
HNC: head and neck cancer
HNSCC: head and neck squamous cell carcinoma
CCRT: concurrent chemoradiotherapy
TPS: tumor proportion score
PFS: progression-free survival
OS: overall survival
CR: complete response
PR: partial responses
SD: stable disease
PD: progressive disease

## References

[1] Cooper JS, Porter K, Mallin K, Hoffman HT, Weber RS, Ang KK, Gay EG, Langer CJ (2009). National Cancer Database report on cancer of the head and neck: 10-year update. Head Neck.

[2] Carvalho AL, Nishimoto IN, Califano JA, Kowalski LP (2005). Trends in incidence and prognosis for head and neck cancer in the United States: a site-specific analysis of the SEER database. Int J Cancer.

[3] Budach W, Bölke E, Kammers K, Gerber PA, Orth K, Gripp S, Matuschek C (2016). Induction chemotherapy followed by concurrent radio-chemotherapy versus concurrent radio-chemotherapy alone as treatment of locally advanced squamous cell carcinoma of the head and neck (HNSCC): a meta-analysis of randomized trials. Radiother Oncol.

[4] Lefebvre JL, Andry G, Chevalier D, Luboinski B, Collette L, Traissac L, de Raucourt D, Langendijk JA, EORTC Head and Neck Cancer Group (2012). Laryngeal preservation with induction chemotherapy for hypopharyngeal squamous cell carcinoma: 10-year results of EORTC trial 24891. Ann Oncol.

[5] Henriques De Figueiredo B, Fortpied C, Menis J, Lefebvre JL, Barzan L, de Raucourt D, Geoffrois L, Giurgea L, Hupperets P, Leemans CR, Licitra L, Rolland F, Tesselaar M (2016). Long-term update of the 24954 EORTC phase III trial on larynx preservation. Eur J Cancer.

[6] Cognetti F, Pinnarö P, Ruggeri EM, Carlini P, Perrino A, Impiombato FA, Calabresi F, Chilelli MG, Giannarelli D (1989). Prognostic factors for chemotherapy response and survival using combination chemotherapy as initial treatment of advanced head and neck squamous cell cancer. J Clin Oncol.

[7] Montero EH, Trufero JM, Romeo JA, Terré FC (2008). Comorbidity and prognosis in advanced hypopharyngeal-laryngeal cancer under combined therapy. Tumori.

[8] Karpathiou G, Giroult JB, Forest F, Fournel P, Monaya A, Froudarakis M, Dumollard JM, Prades JM, Gavid M, Peoc'h M (2016). Clinical and histologic predictive factors of response to induction chemotherapy in head and neck squamous cell carcinoma. Am J Clin Pathol.

[9] Karpathiou G, Casteillo F, Giroult JB, Forest F, Fournel P, Monaya A, Froudarakis M, Dumollard JM, Prades JM, Peoc'h M (2016). Prognostic impact of immune microenvironment in laryngeal and pharyngeal squamous cell carcinoma: immune cell subtypes, immuno-suppressive pathways and clinicopathologic characteristics. Oncotarget.

[10] Pardoll DM (2012). The blockade of immune checkpoints in cancer immunotherapy. Nat Rev Cancer.

[11] Rizvi NA, Mazières J, Planchard D, Stinchcombe TE, Dy GK, Antonia SJ, Horn L, Lena H, Minenza E, Mennecier B, Otterson GA, Campos LT, Gandara DR (2015). Activity and safety of nivolumab, an anti-PD-1 immune checkpoint inhibitor, for patients with advanced, refractory squamous non-small-cell lung cancer (CheckMate 063): a phase 2, single-arm trial. Lancet Oncol.

[12] Weber JS, D’Angelo SP, Minor D, Hodi FS, Gutzmer R, Neyns B, Hoeller C, Khushalani NI, Miller WH, Lao CD, Linette GP, Thomas L, Lorigan P (2015). Nivolumab versus chemotherapy in patients with advanced melanoma who progressed after anti-CTLA-4 treatment (CheckMate 037): a randomised, controlled, open-label, phase 3 trial. Lancet Oncol.

[13] Hodi FS, O'Day SJ, McDermott DF, Weber RW, Sosman JA, Haanen JB, Gonzalez R, Robert C, Schadendorf D, Hassel JC, Akerley W, van den Eertwegh AJ, Lutzky J (2010). Improved survival with ipilimumab in patients with metastatic melanoma. N Engl J Med.

[14] Sobin LH, Gospodarowicz MK, Wittekind CH (2009). TNM Classification of Malignant Tumours.

[15] Taube JM, Anders RA, Young GD, Xu H, Sharma R, McMiller TL, Chen S, Klein AP, Pardoll DM, Topalian SL, Chen L (2012). Colocalization of inflammatory response with B7-h1 expression in human melanocytic lesions supports an adaptive resistance mechanism of immune escape. Sci Transl Med.

[16] Tokito T, Azuma K, Kawahara A, Ishii H, Yamada K, Matsuo N, Kinoshita T, Mizukami N, Ono H, Kage M, Hoshino T (2016). Predictive relevance of PD-L1 expression combined with CD8+ TIL density in stage III non-small cell lung cancer patients receiving concurrent chemoradiotherapy. Eur J Cancer.

[17] Barry M, Bleackley RC (2002). Cytotoxic T lymphocytes: all roads lead to death. Nat Rev Immunol.

[18] Fridman WH, Pages F, Sautes-Fridman C, Garon J (2012). The immune contexture in human tumours: impact on clinical outcome. Nat Rev Cancer.

[19] Kim HR, Ha SJ, Hong MH, Heo SJ, Koh YW, Choi EC, Kim EK, Pyo KH, Jung I, Seo D, Choi J, Cho BC, Yoon SO (2016). PD-L1 expression on immune cells, but not on tumor cells, is a favorable prognostic factor for head and neck cancer patients. Sci Rep.

[20] Balermpas P, Rödel F, Rödel C, Krause M, Linge A, Lohaus F, Baumann M, Tinhofer I, Budach V, Gkika E, Stuschke M, Avlar M, Grosu AL (2016). CD8+ tumour-infiltrating lymphocytes in relation to HPV status and clinical outcome in patients with head and neck cancer after postoperative chemoradiotherapy: a multicentre study of the German cancer consortium radiation oncology group (DKTK-ROG). Int J Cancer.

[21] Balermpas P, Michel Y, Wagenblast J, Seitz O, Weiss C, Rödel F, Rödel C, Fokas E (2014). Tumour-infiltrating lymphocytes predict response to definitive chemoradiotherapy in head and neck cancer. Br J Cancer.

[22] Kershaw MH, Teng MW, Smyth MJ, Dancy PK (2005). Supernatural T cells: genetic modification of T cells for cancer therapy. Nat Rev Immunol.

[23] Galluzzi L, Senovilla L, Zitvogel L, Kroemer G (2012). The secret ally: immunostimulation by anticancer drugs. Nat Rev Drug Discov.

[24] Kang TH, Mao CP, Lee SY, Chen A, Lee JH, Kim TW, Alvarez RD, Roden RB, Pardoll D, Hung CF, Wu TC (2013). Chemotherapy acts as an adjuvant to convert the tumor microenvironment into a highly permissive state for vaccination-induced antitumor immunity. Cancer Res.

[25] Lim YJ, Koh J, Kim K, Chie EK, Kim B, Lee KB, Jang JY, Kim SW, Oh DY, Bang YJ, Ha SW (2015). High ratio of programmed cell death protein 1 (PD-1)(+)/CD8(+) tumor-infiltrating lymphocytes identifies a poor prognostic subset of extrahepatic bile duct cancer undergoing surgery plus adjuvant chemoradiotherapy. Radiother Oncol.

[26] Hsu MC, Hsiao JR, Chang KC, Wu YH, Su IJ, Jin YT, Chang Y (2010). Increase of programmed death-1-expressing intratumoral CD8 T cells predicts a poor prognosis fornasopharyngealcarcinoma. Mod Pathol.

[27] Ferris RL, Blumenschein G, Fayette J, Guigay J, Colevas AD, Licitra L, Harrington K, Kasper S, Vokes EE, Even C, Worden F, Saba NF, Iglesias Docampo LC (2016). Nivolumab for recurrent squamous-cell carcinoma of the head and neck. N Engl J Med.

[28] Mu CY, Huang JA, Chen Y, Chen C, Zhang XG (2011). High expression of PD-L1 in lung cancer may contribute to poor prognosis and tumor cells immune escape through suppressing tumor infiltrating dendritic cells maturation. Med Oncol.

[29] Chen YB, Mu CY, Huang JA (2012). Clinical significance of programmed death-1 ligand-1 expression in patients with non-small cell lung cancer: a 5-year-follow-up study. Tumori.

[30] Shin SJ, Jeon YK, Kim PJ, Cho YM, Koh J, Chung DH, Go H (2016). Clinicopathologic analysis of PD-L1 and PD-L2 expression in renal cell carcinoma: association with oncogenic proteins status. Ann Surg Oncol.

[31] Schalper KA, Velcheti V, Carvajal D, Wimberly H, Brown J, Pusztai L, Rimm DL (2014). In situ tumor PD-L1 mRNA expression is associated with increased tils and better outcome in breast carcinomas. Clin Cancer Res.

[32] Lin YM, Sung WW, Hsieh MJ, Tsai SC, Lai HW, Yang SM, Shen KH, Chen MK, Lee H, Yeh KT, Chen CJ (2015). High PD-L1 expression correlates with metastasis and poor prognosis in oral squamous cell carcinoma. PLoS One.

[33] Motoshima T, Komohara Y, Ma C, Dewi AK, Noguchi H, Yamada S, Nakayama T, Kitada S, Kawano Y, Takahashi W, Sugimoto M, Takeya M, Fujimoto N (2017). PD-L1 expression in papillary renal cell carcinoma. BMC Urol.

[34] Park IH, Kong SY, Ro JY, Kwon Y, Kang JH, Mo HJ, Jung SY, Lee S, Lee KS, Kang HS, Lee E, Joo J, Ro J (2016). Prognostic implications of tumor-infiltrating lymphocytes in association with programmed death ligand 1 expression in early-stage breast cancer. Clin Breast Cancer.

[35] Vassilakopoulou M, Avgeris M, Velcheti V, Kotoula V, Rampias T, Chatzopoulos K, Perisanidis C, Kontos CK, Giotakis AI, Scorilas A, Rimm D, Sasaki C, Fountzilas G (2016). Evaluation of PD-L1 expression and associated tumor-infiltrating lymphocytes in laryngeal squamous cell carcinoma. Clin Cancer Res.

[36] Chang AM, Chiosea SI, Altman A, Pagdanganan HA, Ma C (2017). Programmed death-ligand 1 expression, microsatellite instability, epstein-barr virus, and human papillomavirus in nasopharyngeal carcinomas of patients from the Philippines. Head Neck Pathol.

[37] Koh J, Ock CY, Kim JW, Nam SK, Kwak Y, Yun S, Ahn SH, Park DJ, Kim HH, Kim WH, Lee HS (2017). Clinicopathologic implications of immune classification by PD-L1 expression and CD8-positive tumor-infiltrating lymphocytes in stage II and III gastric cancer patients. Oncotarget.

[38] Teng MW, Ngiow SF, Ribas A, Smyth MJ (2015). Classifying cancers based on T-cell infiltration and PD-L1. Cancer Res.

[39] Herbst RS, Soria JC, Kowanetz M, Fine GD, Hamid O, Gordon MS, Sosman JA, McDermott DF, Powderly JD, Gettinger SN, Kohrt HE, Horn L, Lawrence DP (2014). Predictive correlates of response to the anti-PD-L1 antibody MPDL3280A in cancer patients. Nature.

[40] Roach C, Zhang N, Corigliano E, Jansson M, Toland G, Ponto G, Dolled-Filhart M, Emancipator K, Stanforth D, Kulangara K (2016). Development of a companion diagnostic PD-L1 immunohistochemistry assay for pembrolizumab therapy in non-small-cell lung cancer. Appl Immunohistochem Mol Morphol.

[41] Langer CJ, Gadgeel SM, Borghaei H, Papadimitrakopoulou VA, Patnaik A, Powell SF, Gentzler RD, Martins RG, Stevenson JP, Jalal SI, Panwalkar A, Yang JC, Gubens M (2016). Carboplatin and pemetrexed with or without pembrolizumab for advanced, non-squamous non-small-cell lung cancer: a randomised, phase 2 cohort of the open-label KEYNOTE-021 study. Lancet Oncol.

[42] Yu H, Batenchuk C, Badzio A, Boyle TA, Czapiewski P, Chan DC, Lu X, Gao D, Ellison K, Kowalewski AA, Rivard CJ, Dziadziuszko R, Zhou C (2017). PD-L1 expression by two complementary diagnostic assays and mRNA in situ hybridization in small cell lung cancer. J Thorac Oncol.

[43] Lee HJ, Kim JY, Park IA, Song IH, Yu JH, Ahn JH, Gong G (2015). Prognostic prognostic significance of tumor-infiltrating lymphocytes and the tertiary lymphoid structures in HER2-positive breast cancer treated with adjuvant trastuzumab. Am J Clin Pathol.

[44] Garrido F, Perea F, Bernal M, Sánchez-Palencia A, Aptsiauri N, Ruiz-Cabello F (2017). The escape of cancer from T cell-mediated immune surveillance: HLA class I loss and tumor tissue architecture. Vaccines (Basel).

