# Supplementary material for: Association between PD-L1 expression combined with tumor-infiltrating lymphocytes and the prognosis of patients with advanced hypopharyngeal squamous cell carcinoma
Source: Oncotarget. 2017 Oct 6;8(54):92699–714. doi: 10.18632/oncotarget.21564 (PMC5696215; doi:10.18632/oncotarget.21564)
Supplement: Supplementary file 1 [file oncotarget-08-92699-s001.pdf]

Association between PD-L1 expression combined with tumor-infiltrating lymphocytes and the prognosis of patients with advanced hypopharyngeal squamous cell carcinoma

SUPPLEMENTARY MATERIALS

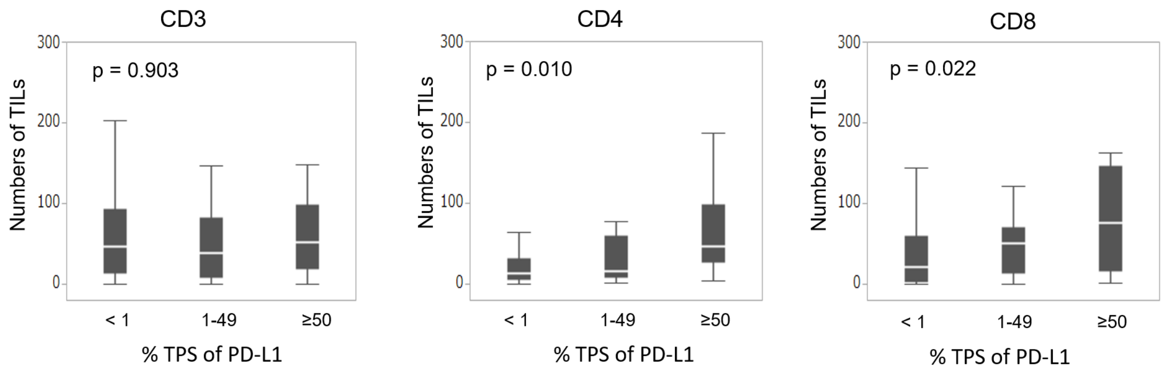

Supplementary Figure 1: Numbers of CD3^{+}, CD4^{+}, and CD8^{+} TILs in tumor samples with < 1%, 1%–49%, and ≥ 50% TPS PD-L1 expression are shown. Significant differences were evaluated using a Kruskal-Wallis test.

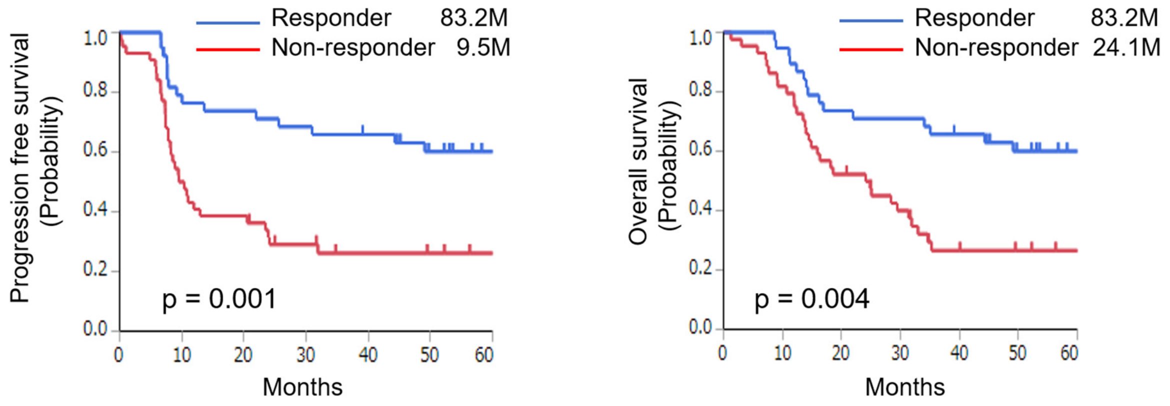

Supplementary Figure 2: Kaplan-Meier analysis of PFS **(A)** and OS **(B)** in NAC responders and non-responders. Significant differences were evaluated using a log-rank test.
